# Single-Scan Heteronuclear ^13^C–^15^N *J*-Coupling NMR Observations Enhanced by Dissolution Dynamic Nuclear Polarization

**DOI:** 10.1021/acs.jpclett.4c01190

**Published:** 2024-05-20

**Authors:** Kawarpal Singh, Lucio Frydman

**Affiliations:** † Department of Chemical and Biological Physics, Weizmann Institute of Science, 7610001 Rehovot, Israel; ‡ Yusuf Hamied Department of Chemistry, University of Cambridge, Lensfield Road, CB2 1EW Cambridge, United Kingdom

## Abstract

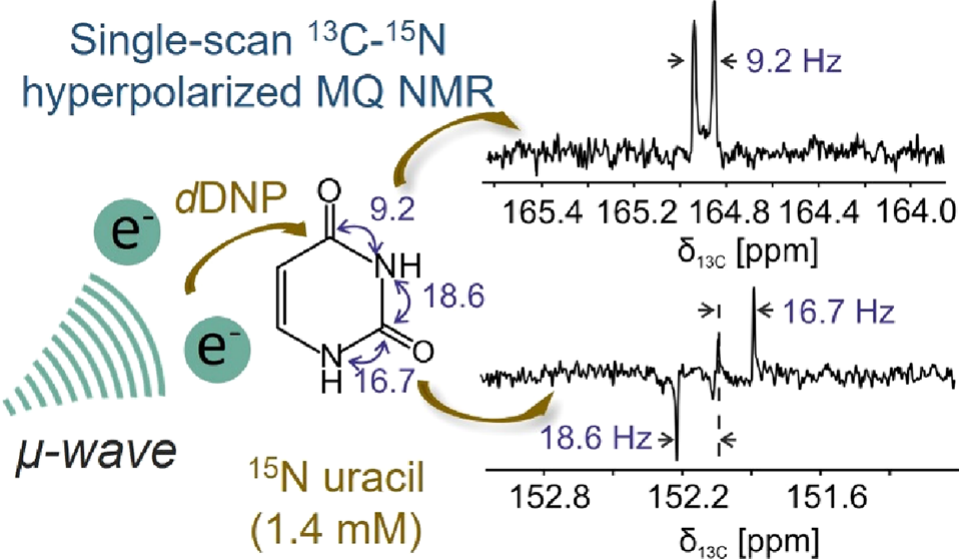

Heteronuclear ^13^C–^15^N couplings were measured in single-scan nuclear magnetic resonance (NMR) experiments for a variety of nitrogen-containing chemical compounds with varied structural characteristics, by using a one-dimensional (1D) ^13^C–^15^N multiple-quantum (MQ)-filtered experiment. Sensitivity limitations of the MQ filtering were overcome by the combined use of ^15^N labeling and dissolution dynamic nuclear polarization (*d*DNP), performed at cryogenic conditions and followed by quick and optimized sample melting and transfer procedures. Coupling information could thus be obtained from nucleotide bases, amino acids, urea, and aliphatic and aromatic amides, including the measurement of relatively small *J*-couplings directly from the 1D filtered spectra. This experiment could pave the way for NMR-based analytical applications that investigate structural and stereochemical insights into nitrogen-containing compounds, including dipeptides and proteins, while relying on heteronuclear couplings and nuclear hyperpolarization.

Nuclear magnetic resonance (NMR) spectroscopy is commonly used for analyzing and determining the structures of organic and pharmaceutical molecules.^1^ Multidimensional NMR studies based on various polarization transfer and coherence selection strategies are usually employed for this,^2,3^ with *J*-couplings between nuclei often delivering the sought information about connectivities and chemical structure. The extent of *J*-couplings is determined by the type of nuclei and their location in a molecule. Although direct realization of interatomic connectivity within a molecule can be obtained via these *J*-couplings,^4^ this may become difficult for conventional organic molecules when dealing with nuclei having low natural abundance. ^13^C–^13^C connectivities have been shown to be most informative when interrogated with the Incredible Natural Abundance DoublE QUAntum Transfer Experiment (INADEQUATE).^5−7^ In biomolecular and natural product NMR studies, however, it is heteronuclear correlations between ^13^C and ^15^N that have often been proven to be the most informative for studying the structures and chemical transformations of monocyclic and fused nitrogen heterocycles, natural products,^8^ drug screening, and small molecules of ambiguous regiochemistry.^9^ To investigate these ^13^C–^15^N couplings various strategies have been used, including two-dimensional (2D) heteronuclear multiple-bond correlation (HMBC) for multiple-bond H–(C)–N correlation experiments using single- or multiple-bond ^*n*^*J*_CN_ coupling constants.^10−14^

Because of the low natural abundances of ^15^N (0.365%) and ^13^C (1.1%), however, both homo- and heteronuclear links are difficult to establish at low concentrations. It has been recently shown that INADEQUATE-like measurements can be carried out at low concentrations, in natural abundance and in a single shot, by using hyperpolarized solutions.^15^ The most general way to obtain such hyperpolarized solutions is by relying on dissolution dynamic nuclear polarization (*d*DNP), an experiment capable of enhancing ^13^C solution-state NMR sensitivity by over 4 orders of magnitude.^16,17^ In this Letter, we show that *d*DNP-enhanced one-dimensional (1D) ^13^C–^15^N multiple-quantum (MQ)-filtered spectra can also be collected within a single shot for a range of nitrogen-containing chemical compounds having different structural features, including nucleotide bases, amino acids, urea, and aliphatic and aromatic amides. Carbon–nitrogen couplings were used to filter quaternary and methylene signals bonded to nitrogen at low concentrations and over a range of ^13^C *T*_1_ relaxation times. Signal enhancements exceeding 1000× were obtained in these single-scan 1D ^13^C–^15^N MQ NMR acquisitions of the *d*DNP-enhanced substrates, delivering spectra containing structural information about the compounds. Carbon–nitrogen connectivities were assessed here for urea, glycine, formamide, benzamide, and uracil, using *J*_CN_-driven correlations in conjunction with the enhancements brought about by *d*DNP.

*d*DNP provides a general approach to low-γ nuclei hyperpolarization^18^ by relying on irradiating with microwaves a frozen glass containing the targeted substrates with radicals comixed in a glass-forming solvent, placed in a magnetic field at a temperature of ∼1 K.^19−22^ As a result of the irradiation, the very high electron spin polarization that is achieved under such conditions will transfer over the course of minutes or hours to the surrounding nuclear spins, including the ^13^C that were here targeted. In order to perform a solution-state NMR experiment, the sample is then quickly melted and dissolved using a superheated, pressurized solvent, which transferred it to a 5 mm NMR tube that was waiting within the NMR magnet. The desired MQ-edited NMR spectra were recorded after stabilizing the solution, producing thousands-fold signal increases for slowly decaying species like ^13^C. As the post-dissolution spin polarization decays by longitudinal relaxation, the type of nuclei that can profit from the *d*DNP methodology depends on the targeted nuclei *T*_1_ times and the rapidity of the transfer process. For simplicity all experiments were of a 1D nature, and MQ-based 2D experiments that would demand single-scan ultrafast NMR^23−25^ were not assayed. Although the spin hyperpolarization/dissolution procedure was a single, irreversible step, acquiring these *J*-edited data offered the option to acquire simple spectra with high resolution. This stands in contrast to the alternative collection of a 2D HMBC/HMQC-like experiment, which although more onerous sensitivity-wise would have provided a richer information.

Provisions were taken here to achieve good repeatability through effective sample handling and optimized chase pressures, leading to short transfer times that enabled the observation of protonated and nonprotonated carbon signals. By injecting stable, repeatable post-dissolution volumes (0.5 mL), the hyperpolarized samples could deliver the ∼1 Hz line shapes required to record a well-resolved double quantum spectrum without phase cycling. To do so, samples were dissolved in either 4 mL of D_2_O for water-based dissolutions or 4.5 mL of methanol for non-aqueous dissolutions. In all cases, solvents were superheated until they achieved ca. 10 bar; for the methanol-based dissolutions, a direct injection setup was used with the helium gas chase pressure and time tuned to 10 bar and 1 s, respectively; this delivered a rapid injection with a clear solution in ca. 2 s. An Arduino-controlled rapid injection mechanism^26,27^ was used for the water-based dissolutions, producing steady, bubble-free injections within 3 s of dissolution.

In all cases, the 1D ^13^C-detected MQ-filtered experiments^28,29^ were completed using a Bruker TCI Prodigy instrument, a proton-optimized triple resonance NMR “inverse” probe, and an 11.7 T Magnex magnet interfaced to a Bruker Neo console. Although the combined sensitivity of the probe and the *d*DNP sufficed to detect ^13^C–^13^C MQ-filtered data at natural abundance and in a single scan, the lower abundance of the ^15^N species demanded the use of labeled compounds. Each 1D hyperpolarized NMR experiment began immediately after the hyperpolarized material was injected into the 5 mm tube waiting inside the magnet bore. The pulse sequence used in these experiments was the 1D ^13^C–^15^N MQ-filtered version shown in Figure 1, resulting in spectra showing only isolated ^13^C–^15^N spin pairs involving C–(N) and (N)–C–(N) spin systems. The multiplets arising from these systems will be centered at the offset of the ^13^C, and their amplitudes will be modulated depending upon the τ-delay used. Spectra were obtained by assuming that signals associated with isolated C–(N) spin pairs had a sin(π*J*_CN_τ) modulation, and those of (N)–C–(N) spin systems had a sin(π*J*_CN_τ)cos(π*J*_CN_τ) modulation.^30^ For samples featuring a single C–(N) spin system, namely benzamide, formamide, and glycine, the τ-delay was thus simply set to 1/2*J*_CN_; for uracil, comprising both C–(N) and (N)–C–(N) spin systems, the τ-delay was chosen as a compromise yielding the desired signals with good enhancement for both spin systems in a single experiment. A similar τ-delay was used to collect the antiphase urea signal doublet; although this provided solely 65% of the maximum potential signal for this (N)–C–(N) spin system, we considered it a suitable example for testing the kind of sensitivities achievable when dealing with spin systems of unknown coupling constants, where determining the optimized τ-delay a priori would be challenging.

**Figure 1:**
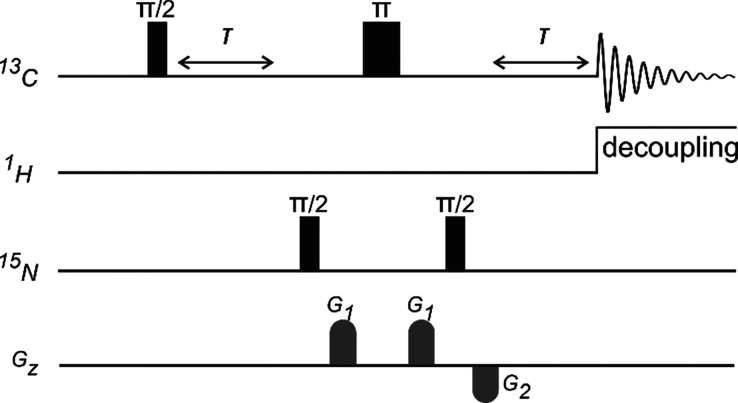
Schematic representation of the pulse sequence used to obtain the 1D ^13^C–^15^N {^1^H}-coupled MQ-filtered *d*DNP spectra. Both *G*_1_ and *G*_2_ gradients along the *z*-direction were of the same length of 1 ms, while the gradient strength of *G*_2_ was 0.8 times that of *G*_1_ (50 G/cm). WALTZ65 decoupling was used to decouple the protons. Each FID was obtained in 1 s. The τ-delay was set for each sample as described in the text.

The central peaks arising from uncoupled ^13^C spins were dephased using gradients *G*_1_ and *G*_2_, where *G*_2_ = 0.8*G*_1_ = 40 G/cm, and all coherence selection gradients were set to 1 ms. The protons were decoupled by the heteronuclear WALTZ65 during the acquisition. All data were processed in MestReNova 14.0 (Mestrelab Research S.L., Santiago de Compostela, Spain). The final post-dissolution concentrations were calculated by using calibration curves obtained from ^1^H signal intensities of solutes, which involved samples prepared in similar concentrations as those arising after DNP post-dissolution.

To execute the *d*DNP experiments, the solutions of ^15^N-labeled 6 M urea and 0.9 M glycine were dissolved in a D_2_O/glycerol glass-forming medium in a ratio of 2:3 and comixed with 10 mM Oxo63 (tris{8-carboxyl-2,2,6,6-tetra[2-(1-hydroxyethyl)]-benzo(1,2-d:4,5-d)bis(1,3)dithiole-4-yl}methyl sodium salt) free radical. DNP-ready 125 mM uracil solutions were prepared in 50:50 TCE/DMSO-*d*_6_ (tetrachloroethylene/dimethyl sulfoxide-*d*_6_) mixtures containing 20 mM BDPA (α,γ-bisdiphenylene-β-phenylallyl) as a polarizing radical. 8 M formamide and 2.8 M benzamide solutions were prepared by dissolving these compounds in mixtures of sulfolane and TCE in 1:1:1 ratios and adding 20 mM of BDPA as a polarizing radical. An Oxford Instruments HyperSense polarizer (3.35 T, Oxford Instruments Molecular Bio Tools, U.K.) was used to hyperpolarize the samples. Along with the Oxford-supplied E2M80 Edwards vacuum pump, an extra EH-500 Edwards booster pump was employed. The working pressure is reduced to 0.4 mbar as a result of this combination, leading to polarizing temperatures in the 1.1–1.6 K range. ^13^C DNP was obtained by irradiating the sample with microwaves at 94.110 GHz for 3–4 h at power levels of 180 mW.

Figure 2a shows the 1D MQ-filtered spectrum of ^15^N-labeled urea obtained in a single scan after hyperpolarization. The spectrum shows a doublet with a separation of ca. 40 Hz, which is as expected for this (N)–C–(N) three-spin system, with the separation between the outer legs of the multiplet reflecting the ^1^*J*_CN_ = 20 Hz single-bond coupling within each pair. The thermally polarized spectrum of this post-dissolution urea sample was hard to obtain due to its relatively low concentration; therefore, a thermally polarized 1D MQ-filtered spectrum of ^15^N-urea at 2 M was obtained with 2048 scans in 14 h (Figure 3a). The thermal MQ-filtered urea spectrum recorded with the same pulse sequence shows a similar multiplicity pattern as the hyperpolarized solution and reveals a *d*DNP enhancement of ∼6000 when compared with the latter. A coupling constant of ^1^*J*_CN_ = 20 Hz could also be confirmed with a conventional 1D ^13^C NMR spectrum of ^13^C–^15^N-urea (Figure S1).

**Figure 2:**
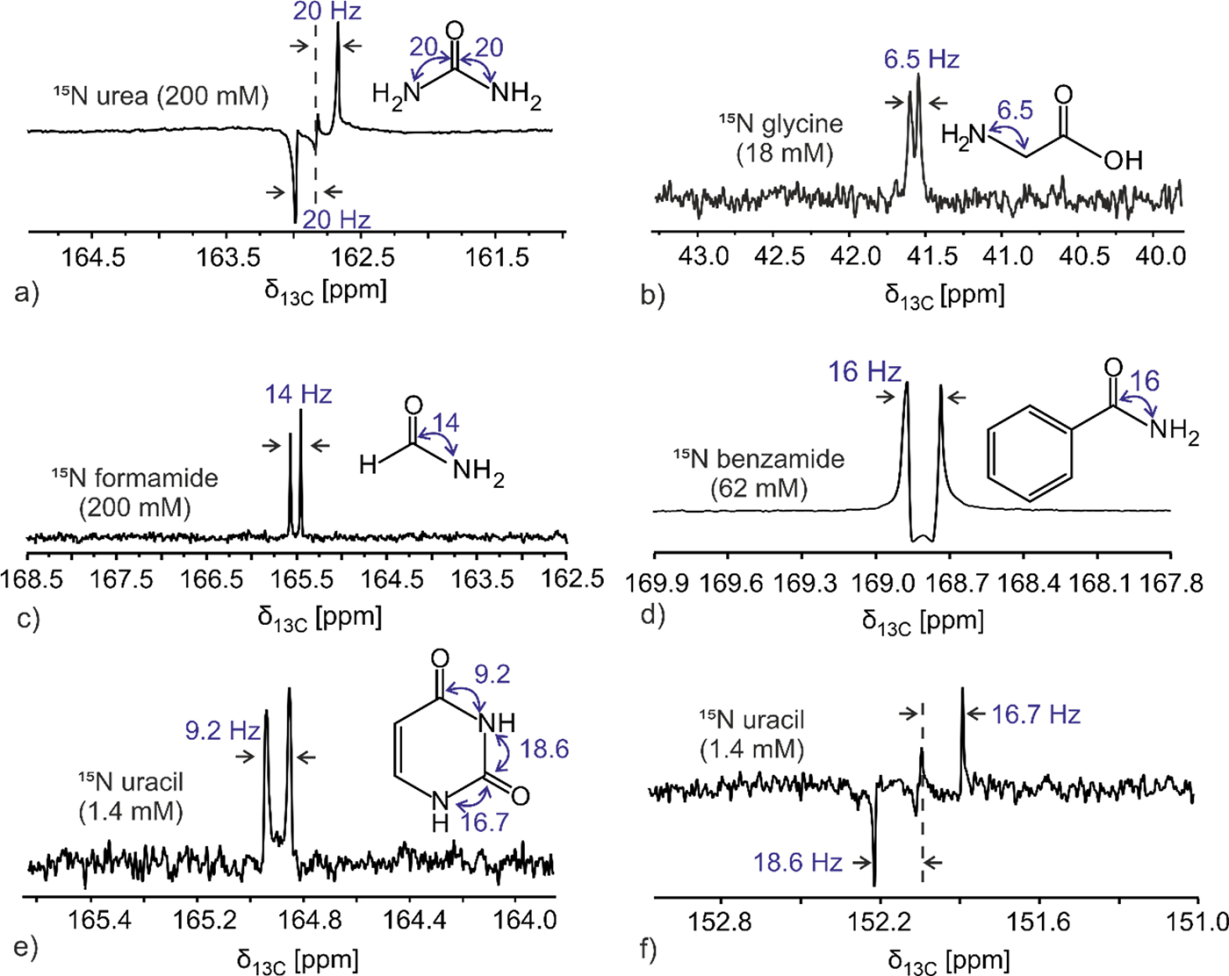
1D hyperpolarized ^13^C–^15^N MQ-filtered spectra acquired in a single scan using *d*DNP-enhanced ^15^N-labeled solutions of (a) urea, (b) glycine, (c) formamide, (d) benzamide, and (e, f) uracil. Spectra were obtained after zero-filling and Fourier transformation only; no window function was applied. Post-dissolution concentrations are given in parentheses.

**Figure 3:**
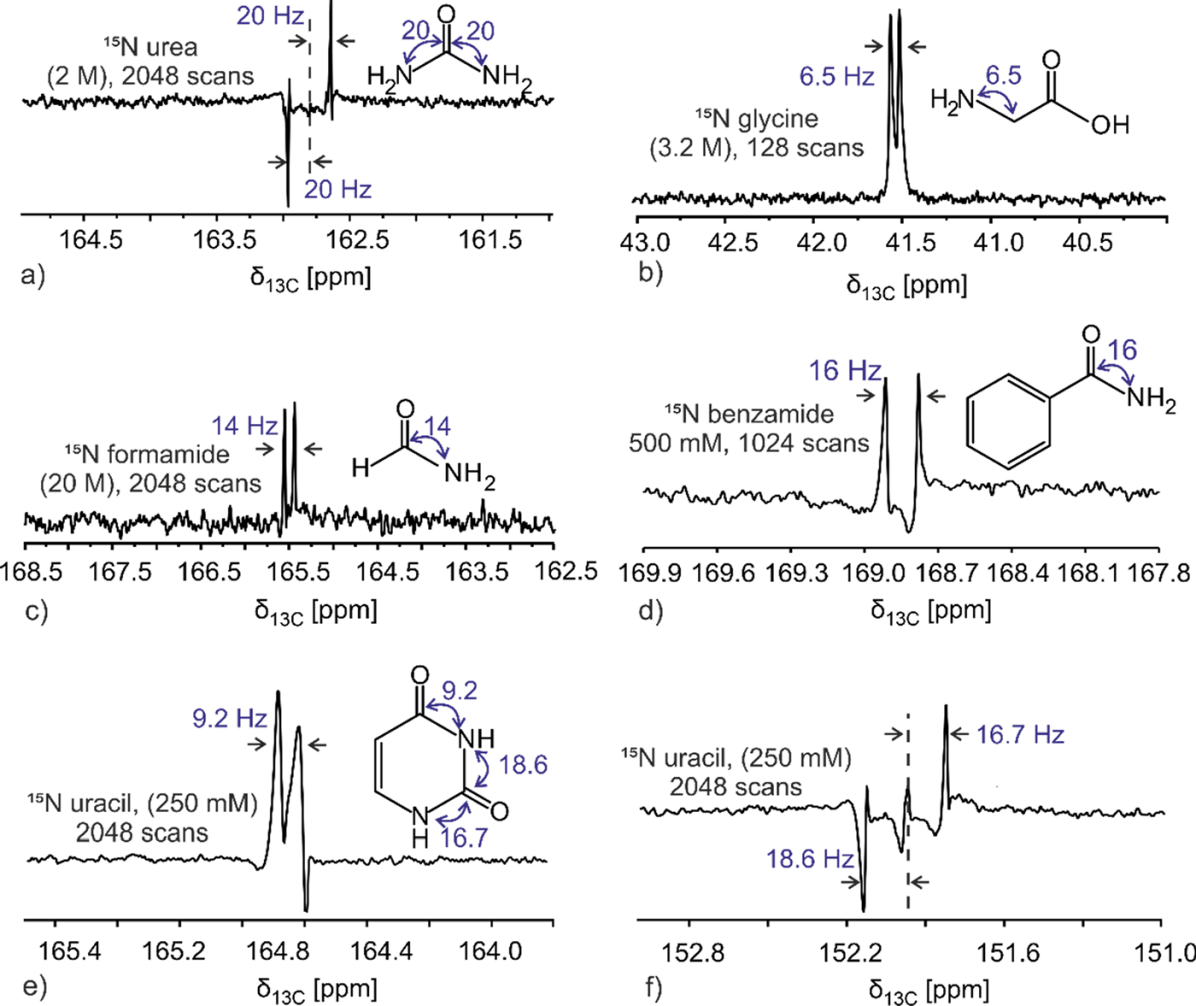
1D thermally polarized ^13^C–^15^N MQ-filtered spectra acquired with concentrated ^15^N-labeled solutions of (a) urea, (b) glycine, (c) formamide, (d) benzamide, and (e, f) uracil. Spectra were obtained after zero-filling and Fourier transformation only; no window function was applied to better illustrate the experimental sensitivity and resolution.

Despite the shorter *T*_1_ relaxation of methylene carbons, the *d*DNP MQ-filtered approach was also tested on glycine. Glycine is also challenged by having a lower solubility than urea, meaning a lower final concentration (18 mM vs 200 mM); it also possesses a smaller *J*_CN_ coupling of 6.5 Hz. Still, a single-scan 1D hyperpolarized ^13^C–^15^N-coupled MQ-filtered spectra of ^15^N-glycine (Figure 2b) provided resolved line shapes with half-widths of <1 Hz, sufficiently sharp to discern the doublet. By comparing the signal-to-noise ratio (SNR) of this hyperpolarized spectrum with that of the thermally polarized ^15^N-glycine spectrum collected on a more concentrated sample (3.2 M) with 128 scans (Figure 3b), an enhancement of ∼1100 is revealed. A coupling constant of 6.5 Hz was also confirmed in a conventional 1D ^13^C NMR spectrum (Figure S2).

Figure 2c shows the 1D hyperpolarized ^13^C–^15^N MQ-filtered NMR spectrum of ^15^N-formamide at 200 mM (Figure 2c), also targeting a protonated carbon with a relatively small *J*_CN_ coupling. In this case, the doublet is also clear, and the spectrum shows an enhancement of ∼8100 in comparison to the thermally polarized spectrum of neat (20 M) ^15^N-formamide obtained with 2048 scans in approximately 14 h (Figure 3c). Analogously, benzamide was investigated in order to examine the aromatic amide system. Although devoid of protons, a smaller final enhancement characterized the carbonyl ^13^C of ^15^N-benzamide (Figure 2d), whose single-scan 1D hyperpolarized ^13^C MQ-filtered spectrum showed a doublet corresponding to a ^1^*J*_CN_ of 16 Hz and an enhancement of ∼3900 vs the 1D thermally polarized spectrum acquired using a more concentrated sample (500 mM) with 1024 scans (7 h, Figure 3d). The 1D conventional ^13^C NMR spectra of ^15^N-acetamide and ^15^N-benzamide, respectively, confirmed the coupling constants of 14 and 16 Hz in both of these compounds (Figures S3 and S4). The final compound examined was a nucleobase, whose low solubility makes it even more challenging to target without hyperpolarization when searching for ^13^C–^15^N coupling information. Still, Figure 2e,f shows that the ^13^C MQ-filtered NMR spectra of hyperpolarized ^15^N-labeled uracil are amenable, even if having a post-dissolution concentration of ∼1.4 mM. A doublet with *J*_CN_ = 9.2 Hz was obtained in the hyperpolarized spectrum between carbonyl carbon C4 and its adjacent nitrogen (Figure 2e). The well resolved line shapes allowed us to distinguish two chemically and magnetically inequivalent nitrogen atoms in the 1D MQ-filtered hyperpolarized spectrum of uracil’s C2 carbon, which evidenced an “antiphase” doublet close to the center of the multiplet that did not cancel out. This incomplete cancelation reflects the fact that, unlike the case arising in urea where both nitrogens are identical and, therefore, the central *J*-components cancel out, the two carbon–nitrogen *J*-couplings in uracil are inequivalent (Figure 2f). The line shapes obtained in the conventional 1D ^13^C NMR spectrum of ^15^N-labeled uracil (Figure S5) also confirm this “doublet-of-doublets” structure. The conventional ^13^C NMR spectrum of ^15^N-uracil also showed another doublet corresponding to C6,with a *J*_CN_ of 10.5 Hz (Figure S5). This was not observed in the hyperpolarized spectrum, presumably due to the relaxation losses affecting such protonated carbon in the post-dissolution period, as well as our relatively low concentrations. As for the carbonyl peaks observable in the MQ-filtered hyperpolarized experiments, these showed an average enhancement of ∼1700 compared to their thermally polarized 1D ^13^C counterparts, as judged from a ^15^N-labeled uracil sample at 250 mM studied using 2048 scans (approximately 14 h acquisition, Figure 3e,f). The detection of ^15^N nuclei could also be considered for studying the compounds in this investigation. This would assist in simplifying multiplet patterns in molecules like uracil, where ^15^N enrichment results in a need to detect both single ^13^C–^15^N spin pairs as well as ^15^N–^13^C–^15^N systems, leading to more complex spectral patterns. On the other hand, ^13^C detection offers an advantage over ^15^N-oriented experiments thanks to the former higher γ factors, enabling the attainment of higher sensitivity. Furthermore, this method can work with more complicated spin systems involving carbon connected to three nitrogen atom (−C(N)_3_−) systems. The signal pattern in these systems is then governed by the *J*-coupled spin pattern for the multispin-1/2 system.

Table S1 summarizes the SNR of the hyperpolarized MQ-filtered spectra with the SNR enhancements obtained against the thermally polarized MQ-filtered NMR counterparts. These enhancement calculations took into account the number of scans and concentrations. The reported improvements are slightly lower than prior *d*DNP studies using other quick injection techniques,^31,32^ presumably due to losses associated with the application of the coherence selection gradients, which may somewhat attenuate the signals after the sudden sample injections. It is interesting to notice the relatively rapid decrease in the hyperpolarization levels that arises as the molecular weight of the targeted compound increases. We hypothesize that these are reflective of the relaxation losses that bigger molecules experience as they transverse regions of low fields, coupled with limitations of the larger molecules to deliver quality glassing media for undergoing an optimized *d*DNP process.

By using hyperpolarized *d*DNP, quality 1D ^13^C–^15^N MQ-filtered spectra providing connectivity and chemical information about carbon and nitrogen atoms in a variety of compounds could be recorded. The method relied on the fast and reliable sample transfer of a hyperpolarized sample, allowing for the acquisition of quality, single-scan sudden dissolution spectra. Careful optimization of the chase pressure and chase time was required for performing these solution injections without bubbles and fluctuations; the speed delivered substantial enhancements even for protonated carbons, with peak widths of ∼1 Hz and center peak accuracies of ∼0.1 Hz. This allowed us to observe *J*-couplings of only a few Hz, such as that of glycine (6.5 Hz). The pre-shimming and tuning using the same NMR tube and mimicking the same composition of solution yet to be obtained with the post-dissolution solution helped in achieving spectra of such quality.

As for the feasibility of the method in general systems, three points need to be considered: (1) the concentration of the sample to be measured, (2) the optimization of the *J*-filtering within the ^13^C–^15^N system, and (3) the degree of hyperpolarization that can be achieved. This work addressed all of these issues. For instance, for the most challenging case here studied (uracil), hyperpolarization was ∼1700 for a ∼1.5 mM concentration. According to the sensitivity of the ensuing spectra, samples with concentrations that are lower by at least 1 order of magnitude would still be measurable with good sensitivity. However, in completely arbitrary systems where the degree of hyperpolarization may be a priori unknown and the *J*-coupling-derived optimizations would have to be carried out using a “best guess” scenario, similar ∼0.1 mM concentrations may lead to an insufficient SNR, particularly if dealing with protonated sites. Indeed,the carbon–nitrogen *J*-couplings can vary from 6 to 20 Hz depending on the chemical bonding and hybridization of the carbon atoms, leading in general to the need of making an "educated guess".^33−35^ Since the nuclear spin hyperpolarization of the post-dissolution samples decays due to the effect of longitudinal relaxation, carbonyl and quaternary carbons that are not attached to protons and have a longer *T*_1_ are usually favored by these experiments. Still, we could investigate a variety of small molecules containing atoms bonded to protons, demonstrating the general usefulness of the experiment for these carbons as well. It should also be remarked that, even if accounting for the very positive aspects of *d*DNP, the time taken to perform a sample injection and the ca. 10-fold dilution occurring post-dissolution result in a considerable drop in sensitivity. I; in some instances, like uracil’s protonated carbon, this prevented us from obtaining the hyperpolarized information altogether. Methods to deal with such problems must be devised. Even with such handicaps, the present study could open a range of NMR-based analytical applications to investigate structural and stereochemical insights in nitrogen-containing compounds through sensitivity increases, including additional dipeptide and protein applications. Furthermore, by employing strategies to decrease dilution and relaxation effects,^36−38^ alternative heteronuclear systems with low natural abundance can be exploited.

## Supporting Information Available

Materials and methods, including additional experimental and data processing details, 1D ^13^C NMR spectra for all compounds, and enhancement calculations table (PDF)

## References

[ref1] Friebolin H. In Basic One- and Two-Dimensional NMR Spectroscopy, 5th ed.; Wiley-VCH, 2010.

[ref2] Gibbs E. B.; Kriwacki R. W. Direct detection of carbon and nitrogen nuclei for high-resolution analysis of intrinsically disordered proteins using NMR spectroscopy. Methods 2018, 138–139, 39–46. 10.1016/j.ymeth.2018.01.004. PMC5984115 29341926

[ref3] Bermel W.; Bertini I.; Felli I. C.; Piccioli M.; Pierattelli R. ^13^C-detected protonless NMR spectroscopy of proteins in solution. Prog. Nucl. Magn. Reson. Spectrosc. 2006, 48, 25–45. 10.1016/j.pnmrs.2005.09.002.

[ref4] Günther H. In NMR Spectroscopy: Basic Principles, Concepts and Applications in Chemistry, 3rd ed.; Wiley-VCH, 2013.

[ref5] Bax A.; Freeman R. Investigation of ^13^C-^13^C couplings in natural abundance samples: The strong coupling case. J. Magn, Reson. 1980, 41, 507–511. 10.1016/0022-2364(80)90309-1.

[ref6] Nielsen N. C.; Thøgersen H.; Sørensen O. W. Doubling the sensitivity of INADEQUATE for tracing out the carbon skeleton of Molecules by NMR. J. Am. Chem. Soc. 1995, 117, 11365–11366. 10.1021/ja00150a045.

[ref7] Bax A.; Freeman R.; Kempsell S. P. Natural abundance carbon-13-carbon-13 coupling observed via double-quantum coherence. J. Am. Chem. Soc. 1980, 102, 4849–4851. 10.1021/ja00534a056.

[ref8] Martin G. E.; Hilton B. D.; Blinov K. A.; Williams A. J. ^13^C-^15^N correlation via unsymmetrical indirect covariance NMR: Application to vinblastine. J. Nat. Prod. 2007, 70, 1966–1970. 10.1021/np070361t. 18044844

[ref9] Chatzopoulou M.; Martínez R. F.; Willis N. J.; Claridge T. D. W.; Wilson F. X.; Wynne G. M.; Davies S. G.; Russell A. J. The Dimroth rearrangement as a probable cause for structural misassignments in imidazo [1,2-a] pyrimidines: A ^15^N-labelling study and an easy method for the determination of regiochemistry. Tetrahedron 2018, 74, 5280–5288. 10.1016/j.tet.2018.06.033.

[ref10] Kline M.; Pierce D.; Cheatham S. Assignment of oxime and hydrazone configuration using ^1^H-^15^N and ^13^C-^15^N coupling measurements at natural abundance. Magn. Reson. Chem. 2017, 55, 154–156. 10.1002/mrc.4536. 27706849

[ref11] Dorn R. W.; Wall B. J.; Ference S. B.; Norris S. R.; Lubach J. W.; Rossini A. J.; VanVeller B. Attached nitrogen test by ^13^C–^14^N solid-state NMR spectroscopy for the structure determination of heterocyclic isomers. Org. Lett. 2022, 24, 5635–5640. 10.1021/acs.orglett.2c01576. 35731042 PMC9933616

[ref12] Cheatham S.; Gierth P.; Bermel W.; Kupče E̅. HCNMBC – A pulse sequence for H–(C)–N Multiple Bond correlations at natural isotopic abundance. J. Magn. Reson. 2014, 247, 38–41. 10.1016/j.jmr.2014.07.011. 25233112

[ref13] Kupče E̅.; Wrackmeyer B. ^13^C detected ^15^N single bond ^13^C coupling measurements at the natural isotopic abundance. J. Magn. Reson. 2017, 279, 68–73. 10.1016/j.jmr.2017.04.015. 28475948

[ref14] Cheatham S.; Kline M.; Kupče E̅. Exploiting natural abundance ^13^C–^15^N coupling as a method for identification of nitrogen heterocycles: practical use of the HCNMBC sequence. Magn. Reson. Chem. 2015, 53, 363–368. 10.1002/mrc.4205. 25594305

[ref15] Otikovs M.; Olsen G. L.; Kupče E̅.; Frydman L. Natural abundance, single-Scan ^13^C-^13^C-based structural elucidations by dissolution DNP NMR. J. Am. Chem. Soc. 2019, 141, 1857–1861. 10.1021/jacs.8b12216. 30648853

[ref16] Eills J.; Budker D.; Cavagnero S.; Chekmenev E.; Elliott S.; Jannin S.; Lesage A.; Matysik J.; Meersmann T.; Prisner T.; Reimer J.; Yang H.; Koptyug I. Spin hyperpolarization in modern magnetic resonance. Chem. Rev. 2023, 123, 1417–1551. 10.1021/acs.chemrev.2c00534. 36701528 PMC9951229

[ref17] Dey A.; Charrier B.; Martineau E.; Deborde C.; Gandriau E.; Moing A.; Jacob D.; Eshchenko D.; Schnell M.; Melzi R.; Kurzbach D.; Ceillier M.; Chappuis Q.; Cousin S.; Kempf J.; Jannin S.; Dumez J.; Giraudeau P. Hyperpolarized NMR metabolomics at natural ^13^C abundance. Anal. Chem. 2020, 92, 14867–14871. 10.1021/acs.analchem.0c03510. 33136383 PMC7705890

[ref18] Abragam A.; Goldman M. Principles of dynamic nuclear polarization. Rep. Prog. Phys. 1978, 41, 395–467. 10.1088/0034-4885/41/3/002.

[ref19] Hilty C.; Kurzbach D.; Frydman L. Hyperpolarized water as universal sensitivity booster in biomolecular NMR. Nat. Protoc. 2022, 17, 1621–1657. 10.1038/s41596-022-00693-8. 35546640 PMC12339186

[ref20] Elliott S. J.; Stern Q.; Ceillier M.; El Daraï T.; Cousin S. F.; Cala O.; Jannin S. Practical dissolution dynamic nuclear polarization. Prog. Nucl. Magn. Reson. Spectrosc. 2021, 126–127, 59–100. 10.1016/j.pnmrs.2021.04.002. 34852925

[ref21] Ardenkjaer-Larsen J. H.; Fridlund B.; Gram A.; Hansson G.; Hansson L.; Lerche M. H.; Servin R.; Thaning M.; Golman K. Increase in signal-to-noise ratio of > 10,000 times in liquid-state NMR. Proc. Natl. Acad. Sci. U. S. A. 2003, 100, 10158–10163. 10.1073/pnas.1733835100. 12930897 PMC193532

[ref22] Wolber J.; Ellner F.; Fridlund B.; Gram A.; Jóhannesson H.; Hansson G.; Lerche M. H.; Månsson S.; Servin R.; Thaning M.; Golman K.; Ardenkjaer-Larsen J. H.; Hansson G. Generating highly polarized nuclear spins in solution using dynamic nuclear polarization. Nucl. Instrum. Methods Phys. Res. Sect. A 2004, 526, 173–181. 10.1016/j.nima.2004.03.171.

[ref23] Frydman L.; Blazina D. Ultrafast two-dimensional nuclear magnetic resonance spectroscopy of hyperpolarized solutions. Nat. Phys. 2007, 3, 415–419. 10.1038/nphys597.

[ref24] Dumez J. N.; Milani J.; Vuichoud B.; Bornet A.; Lalande- Martin J.; Tea I.; Yon M.; Maucourt M.; Deborde C.; Moing A.; Frydman L.; Bodenhausen G.; Jannin S.; Giraudeau P. Hyperpolarized NMR of plant and cancer cell extracts at natural abundance. Analyst 2015, 140, 5860–5863. 10.1039/C5AN01203A. 26215673

[ref25] Giraudeau P.; Shrot Y.; Frydman L. Multiple ultrafast broadband 2D NMR spectra of hyperpolarized natural products. J. Am. Chem. Soc. 2009, 131, 13902–13903. 10.1021/ja905096f. 19743849

[ref26] Olsen G.; Markhasin E.; Szekely O.; Bretschneider C.; Frydman L. Optimizing water hyperpolarization and dissolution for sensitivity-enhanced 2D biomolecular NMR. J. Magn. Reson. 2016, 264, 49–58. 10.1016/j.jmr.2016.01.005. 26920830

[ref27] Bowen S.; Hilty C. Rapid sample injection for hyperpolarized NMR spectroscopy. Phys. Chem. Chem. Phys. 2010, 12, 5766–5770. 10.1039/c002316g. 20442947

[ref28] Bax A.; Griffey R. H.; Hawkins B. L. Correlation of proton and nitrogen-15 chemical shifts by multiple quantum NMR. J. Magn. Reson. 1983, 55, 301–315. 10.1016/0022-2364(83)90241-X.

[ref29] Piantini U.; Sørensen O. W.; Ernst R. R. Multiple quantum filters for elucidating NMR coupling networks. J. Am. Chem. Soc. 1982, 104, 6800–6801. 10.1021/ja00388a062.

[ref30] Sørensen O. W.; Eich G. W.; Levitt M. H.; Bodenhausen G.; Ernst R. R. Product operator formalism for the description of NMR pulse experiments. Prog. Nucl. Magn. Reson. Spectrosc. 1984, 16, 163–192. 10.1016/0079-6565(84)80005-9.

[ref31] Katsikis S.; Marin-Montesinos I.; Pons M.; Ludwig C.; Günther U. L. Improved stability and spectral quality in *ex situ* dissolution DNP using an improved transfer device. Appl. Magn. Reson. 2015, 46, 723–729. 10.1007/s00723-015-0680-5.

[ref32] Bornet A.; Jannin S. Optimizing dissolution dynamic nuclear polarization. J. Magn. Reson. 2016, 264, 13–21. 10.1016/j.jmr.2015.12.007. 26920826

[ref33] Hinton J. F.; Ladner K. H.; Stewart W. E. The ^13^C chemical shift and ^13^C-^15^N coupling constant of formamide in aqueous solutions. J. Magn. Reson. 1973, 12 (1), 90–92. 10.1016/0022-2364(73)90108-X.

[ref34] Fiala R.; Sklenár V. ^13^C-detected NMR experiments for measuring chemical shifts and coupling constants in nucleic acid bases. J. Biomol. NMR. 2007, 39, 153–163. 10.1007/s10858-007-9184-4. 17701076

[ref35] Lipnick R. L.; Fissekis J. D. Use of nitrogen-15-proton and nitrogen-15-carbon-13 coupling constants for the measurement of uracil monoanion tautomerism. J. Org. Chem. 1979, 44, 1627–1630. 10.1021/jo01324a010.

[ref36] Kouřil K.; Kouřilová H.; Bartram S.; Levitt M. H.; Meier B. Scalable dissolution-dynamic nuclear polarization with rapid transfer of a polarized solid. Nat. Commun. 2019, 10, 1733 10.1038/s41467-019-09726-5. 30988293 PMC6465283

[ref37] Harris T.; Bretschneider C.; Frydman L. Dissolution DNP NMR with solvent mixtures: Substrate concentration and radical extraction. J. Magn. Reson. 2011, 211, 96–100. 10.1016/j.jmr.2011.04.001. 21531156 PMC5040482

[ref38] Van Meerten S. G. J.; Janssen G. E.; Kentgens A. P. M. Rapid-melt DNP for multidimensional and heteronuclear high-field NMR experiments. J. Magn. Reson. 2020, 310, 106656 10.1016/j.jmr.2019.106656. 31812888

