# Supplementary material for: Single-Scan Heteronuclear 13C–15N J-Coupling NMR Observations Enhanced by Dissolution Dynamic Nuclear Polarization
Source: J Phys Chem Lett. 2024 May 20;15(21):5659–64. doi: 10.1021/acs.jpclett.4c01190 (PMC11145644; doi:10.1021/acs.jpclett.4c01190)
Supplement: Supplementary file 1 — jz4c01190_si_001.pdf [file jz4c01190_si_001.pdf]

Supplementary Information

Single-Scan Heteronuclear ^{13}C-^{15}N *J*-coupling NMR Observations Enhanced by Dissolution Dynamic Nuclear Polarization

Kawarpal Singh[a,b]* and Lucio Frydman[a]*

[a] *Department of Chemical and Biological Physics, Weizmann Institute of Science, 7610001, Rehovot, Israel*

[b] *Yusuf Hamied Department of Chemistry, University of Cambridge, Lensfield road, CB2 1EW, Cambridge, United Kingdom*

Table of Contents

1. Materials and methods 2

1.1 Materials: 2

1.2 Methods: 2

1.2.1. NMR Spectroscopy 2

1.2.2. Data processing 2

2. Supplementary figures 3

2.1. 1D ^{13}C NMR spectrum of ^{13}C-^{15}N-urea 3

2.2. 1D ^{13}C NMR spectrum of ^{15}N-glycine 3

2.3. 1D ^{13}C NMR spectrum of ^{15}N-formamide 4

2.4. 1D ^{13}C NMR spectrum of ^{15}N-benzamide 4

2.5. 1D ^{13}C NMR spectrum of ^{15}N-Uracil 5

3. Enhancement calculations table 5

1. Materials and methods

1.1 Materials: All the chemicals and solvents were purchased from Sigma-Aldrich and used as received. Due to variety of samples involved, the preparation of DNP juice for each of them varies.

1.2 Methods:

1.2.1. NMR Spectroscopy:

1D ^{13}C-^{15}N experiment: For the 1D ^{13}C-^{15}N experiments, a 1D ^{13}C-^{15}N MQ pulse sequence was used to look at the couplings between carbons directly attached to the nitrogen atoms. Coherence selection gradients of 1 ms were used to select the desired coherence pathway. Waltz65 decoupling scheme was used to decouple the protons attached to nitrogen and carbons. Each hyperpolarized experiment was done in a single scan after a delay of 3 seconds required to stabilize and transfer the solution inside the NMR tube after the dissolution. Recording of the thermal experiments, with the post dissolution samples, was impossible due to very low concentrations, therefore, the thermal experiments were done with the concentrated and labelled samples. Each thermally polarized spectrum was obtained with prior shimming and tuning.

1D ^{13}C NMR experiment: For recording the 1D ^{13}C spectrum, a ^{15}N labelled samples were used in good concentration to see the ^{13}C-^{15}N couplings and other peaks corresponding to remaining carbon atoms not bonded to nitrogen atoms.

1.2.2. Data processing: The data was processed in MestReNova 14.0 (Mestrlab Research, S.L., Santiago de Compostela, Spain).

2. Supplementary figures

2.1. 1D conventional ^{13}C NMR spectrum of ^{13}C-^{15}N-urea

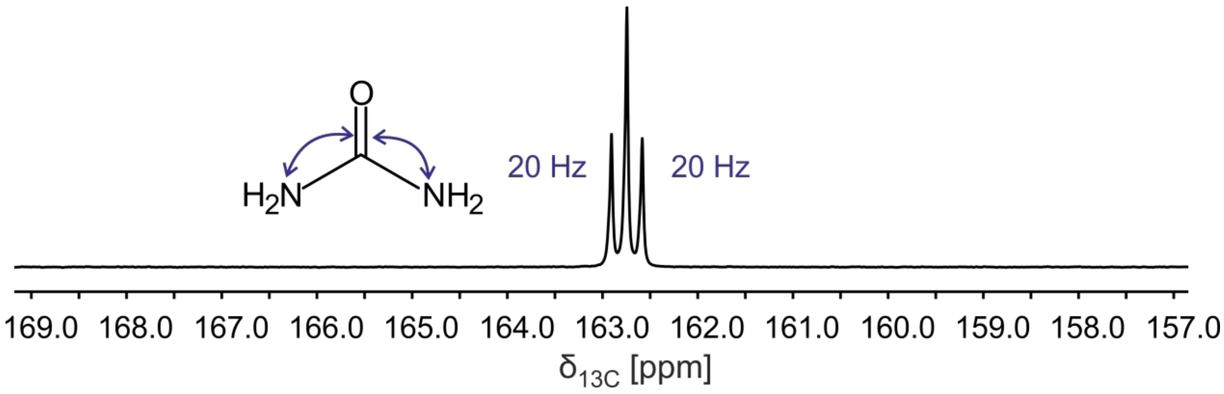

Figure S1: 1D ^{13}C NMR spectrum of ^{13}C, ^{15}N-urea (2 M) in DMSO-d_6 obtained with 4 scans.

2.2. 1D conventional ^{13}C NMR spectrum of ^{15}N-glycine

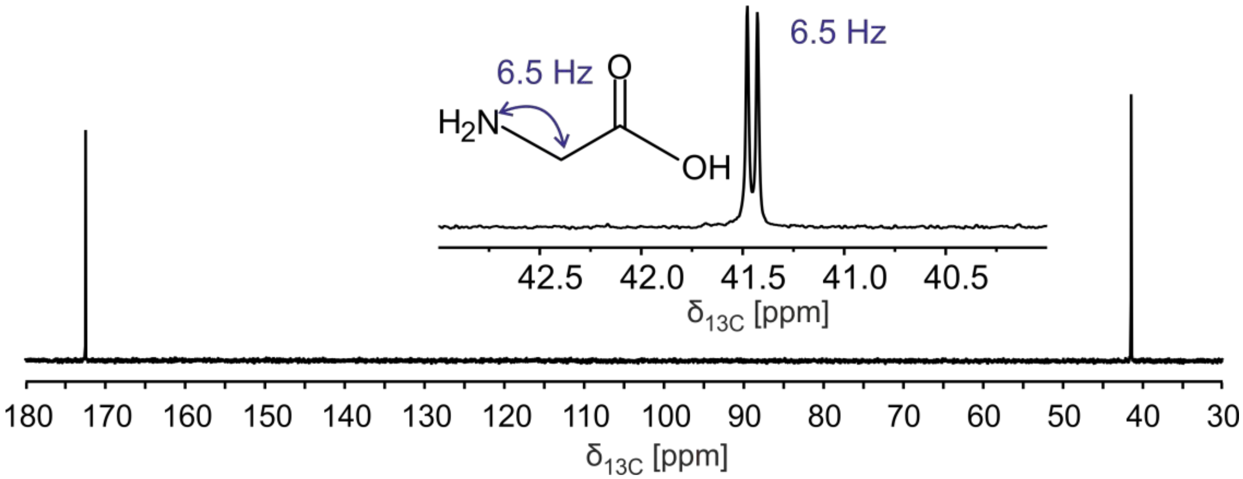

Figure S2: 1D ^{13}C NMR spectrum of ^{15}N- glycine (1.3 M) in D_2O obtained with 64 scans.

2.3. 1D conventional ^{13}C NMR spectrum of ^{15}N-formamide

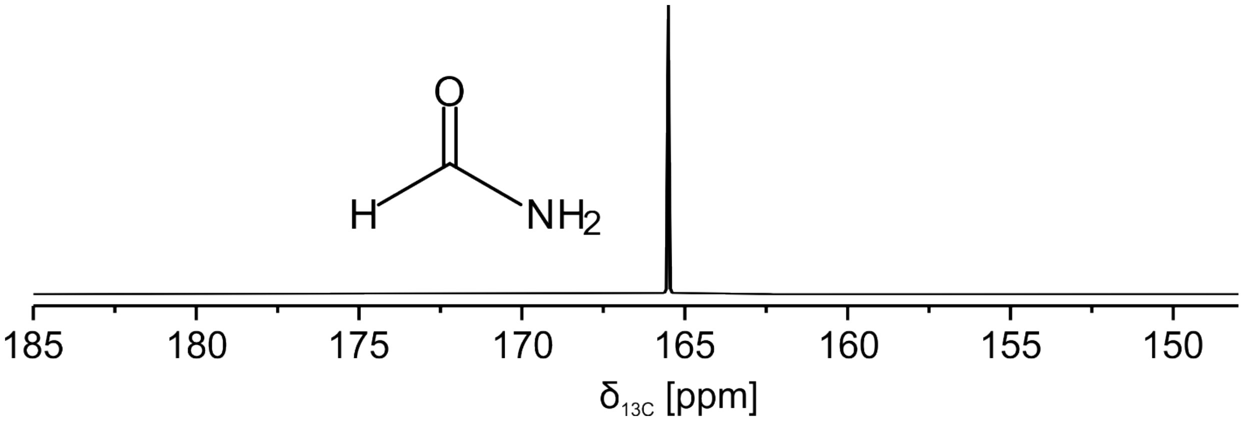

Figure S3: 1D ^{13}C NMR spectrum of formamide (20 M) in DMSO-d_6 obtained with 64 scans.

2.4. 1D conventional ^{13}C NMR spectrum of ^{15}N-benzamide

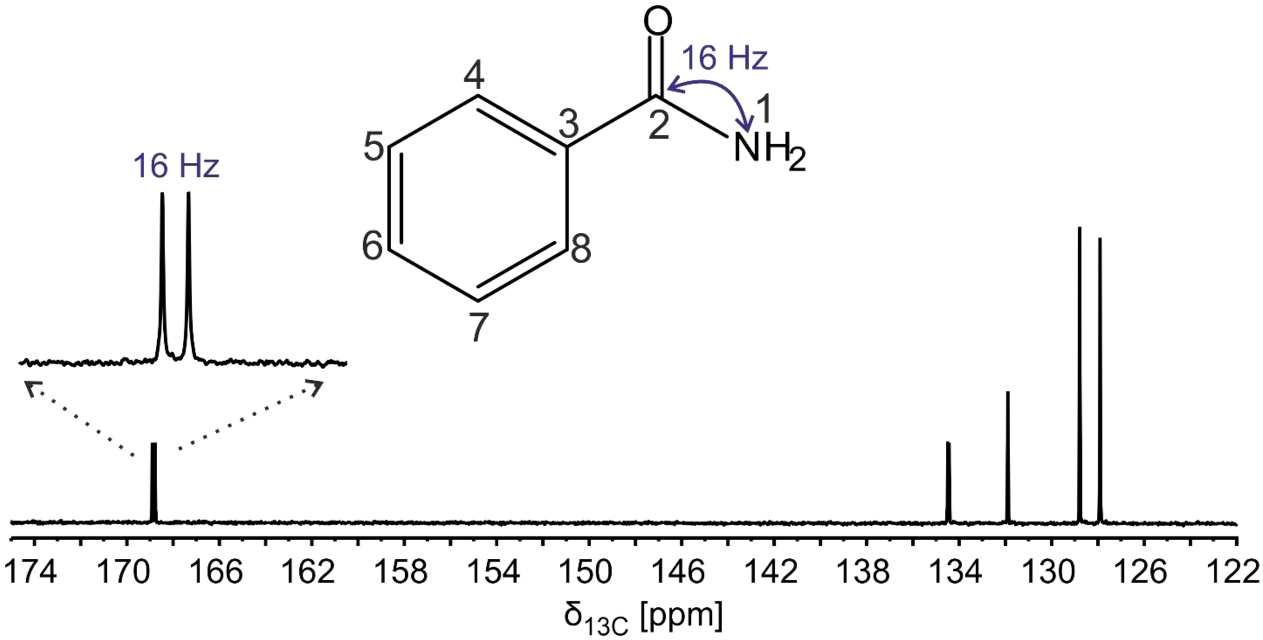

Figure S4: 1D ^{13}C NMR spectrum of ^{15}N-benzamide (500 mM) in DMSO-d_6 obtained with 512 scans.

2.5. 1D conventional ^{13}C NMR spectrum of ^{15}N-Uracil

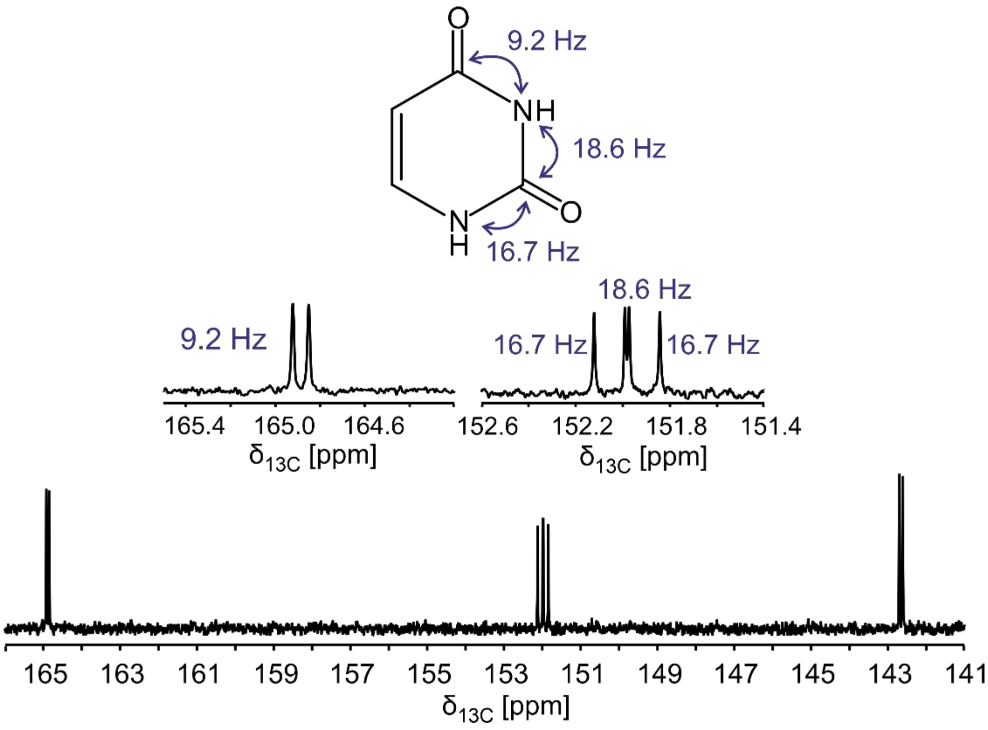

Figure S5: 1D ^{13}C NMR spectrum of ^{15}N-uracil (250 mM) in DMSO-d_6 obtained with 512 scans.

3. Enhancement calculation table

Table S1: Enhancement calculations of hyperpolarized in comparison to thermal polarized MQ-based experiments.

Sample	Relative SNR (dDNP/thermal)	Conc.(thermal/post. diss) in mM	Scan-derived enhancements	dDNP/thermal Enhancements
urea	180/13.8 = 13.1	2000/200 = 10	√2048 = 45.3	5.9 x10^3
glycine	9.5/17 = 0.56	3200/18 = 178	√128 = 11.3	1.1 x10^3
formamide	28.5/15 = 1.9	20000/213= 94	√2048 = 45.3	8.1 x10^3
benzamide	236/15.6 = 15.1	500/62 = 8.1	√1024 = 32	3.9 x10^3
uracil	10.54/50.21 = 0.21	250/1.4 = 178.6	√2048 = 45.3	1.7x10^3
